# Complete genome sequence of *Sphingomonas paucimobilis* AIMST S2, a xenobiotic-degrading bacterium

**DOI:** 10.1038/s41597-019-0289-x

**Published:** 2019-11-25

**Authors:** Suganniiya K. Ravintheran, Sumitra Sivaprakasam, Stella Loke, Su Yin Lee, Ravichandran Manickam, Adibah Yahya, Lawrence Croft, Andrew Millard, Sivachandran Parimannan, Heera Rajandas

**Affiliations:** 1 Centre of Excellence for Omics-Driven Computational Biodiscovery (COMBio), Faculty of Applied Sciences, AIMST University, Bedong, Malaysia; 2 School of Life and Environmental Sciences, Deakin University, Burwood Campus, Burwood, Australia; 3 Biorefinery Technology Laboratory, Department of Biosciences, Faculty of Science, Universiti Teknologi Malaysia, Skudai, Malaysia; 4 Centre for Integrative Ecology, School of Life and Environmental Sciences, Deakin, University, Geelong, Australia; 5 Department of Genetics and Genome Biology, University of Leicester, Leicester, UK

**Keywords:** Sequencing, Genome, Environmental biotechnology, Environmental microbiology

## Abstract

Complete genomes of xenobiotic-degrading microorganisms provide valuable resources for researchers to understand molecular mechanisms involved in bioremediation. Despite the well-known ability of *Sphingomonas paucimobilis* to degrade persistent xenobiotic compounds, a complete genome sequencing is lacking for this organism. In line with this, we report the first complete genome sequence of *Sphingomonas paucimobilis* (strain AIMST S2), an organophosphate and hydrocarbon-degrading bacterium isolated from oil-polluted soil at Kedah, Malaysia. The genome was derived from a hybrid assembly of short and long reads generated by Illumina HiSeq and MinION, respectively. The assembly resulted in a single contig of 4,005,505 bases which consisted of 3,612 CDS and 56 tRNAs. An array of genes involved in xenobiotic degradation and plant-growth promoters were identified, suggesting its’ potential role as an effective microorganism in bioremediation and agriculture. Having reported the first complete genome of the species, this study will serve as a stepping stone for comparative genome analysis of *Sphingomonas* strains and other xenobiotic-degrading microorganisms as well as gene expression studies in organophosphate biodegradation.

## Abstract (summary)

**Table:** 

Measurement(s)	genome • sequence_assembly
Technology Type(s)	DNA sequencing • genome assembly
Sample Characteristic - Organism	Sphingomonas paucimobilis
Sample Characteristic - Environment	oil contaminated soil
Sample Characteristic - Location	Malaysia

Machine-accessible metadata file describing the reported data: 10.6084/m9.figshare.9971711

## Background and Summary

*Sphingomonas* spp. are Gram-negative, oxidase positive and non-fermentative rods^1^. One of the best known species of the genus is *Sphingomonas paucimobilis* as it was originally said to be the only species described in human infection^1,2^. It is a non-spore forming strictly aerobic, yellow-pigmented bacteria that can survive in low nutrient environment^1,3^. *S*. *paucimobilis* is naturally found in diverse environments such as soil and water and also has been shown to have a wide range of xenobiotic-biodegradative abilities^4–6^. Previous studies had shown its’ ability to degrade various types of hydrocarbons and pesticides, specifically chlorpyrifos^7–12^. It is also well recognized for its potential for biofilm formation^13^. Despite the potential role of this bacterium in bioremediation, there is a lack of complete genome in the public domain which will allow for the identification of genes involved in the biodegradation of chlorpyrifos, a widely used organophosphate.

General features of *S*. *paucimobilis* strain AIMST S2 are summarized in Table 1. *S*. *paucimobilis* strain AIMST S2 was first isolated in an oil-contaminated soil sample from Kedah, Malaysia. Following enrichment in LB broth, this strain was acclimatized in M9 minimal medium supplemented with diesel (max. 1% v/v) and chlorpyrifos (max. 100 mg/L) in increasing concentrations, as the sole carbon source. Genomic DNA extraction was performed according to the GeneJet Genomic DNA purification kit’s protocol using a log-phase culture grown in Luria broth. The concentration and quality of extracted DNA was determined using Nanodrop, Qubit dsDNA BR assay and a 1% (v/w) agarose gel. The genomic DNA was then subjected to sequencing via Illumina HiSeq. 2500 and Oxford Nanopore. DNA sequencing was performed with both Illumina and Nanopore technologies as they yield short (~150 bases) and long reads (~10,000 bases), respectively, a combination of which has shown to improve hybrid genome assembly quality by providing accurate, complete genomes without gaps^14^.

**Table 1:** General features of *S*. *paucimobilis* strain AIMST S2 based on MIGS mandatory information.

Items	Description
Investigation type	Bacteria
Project name	Complete genome sequencing of *S*. *paucimobilis* AIMST S2
Latitude and longitude	5.663 N 100.505 E
Geographical location	Malaysia
Collection date	19 December 2008
Isolation source	Oil-contaminated soil
Estimated size	4,005,505 bp
Sequencing method	Illumina HiSeq. 2500 & MinION
Assembly	Hybrid genome assembly (Unicycler)
Assembly level	Complete Genome
Genome representation	Full
Genome coverage	~446.6×
Finishing strategy	Sequencing & assembly

The complete genome sequence reported in this study will be useful for analysis of protein-coding gene families, identification of genomic islands, repeat regions, prophages, and structural rearrangements. Apart from that, the data from this study can be utilized for comparative genome analysis of strains belonging to the genus *Sphingomonas* and other xenobiotic-degrading microorganisms, as well as transcriptome studies of chlorpyrifos biodegradation.

**Fig. 1:**
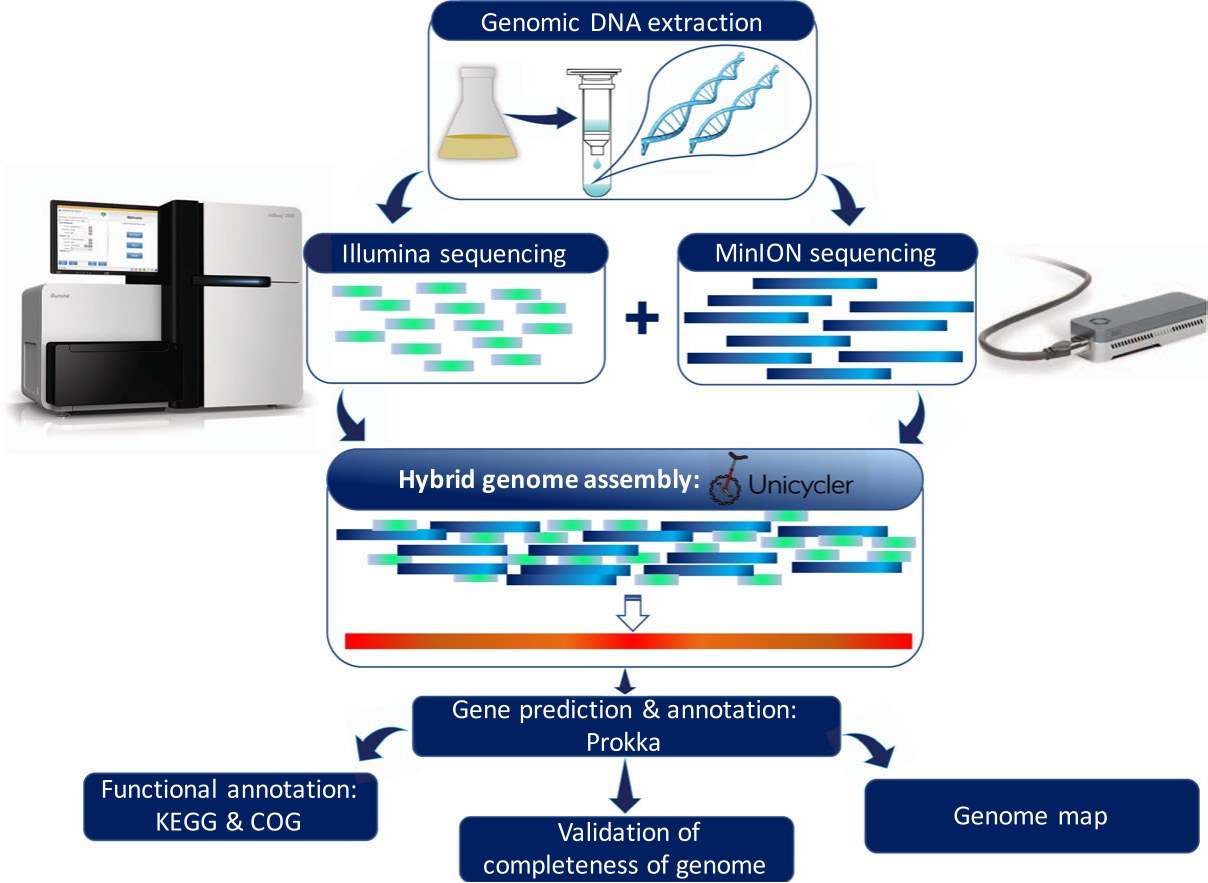
Overview of the experimental design of study.

An overview of the experimental design of the study is illustrated in Fig. 1 and a detailed account of the workflow is provided in the methodology.

## Methods

### Bacterial growth and genomic DNA extraction

*S*. *paucimobilis* was cultivated in LB broth and incubated at 37 °C until it attained an absorbance of ~0.7 at 600 _nm_. The log-phase culture was centrifuged at 10,000 × g for 10 minutes and the cell pellet was subjected to genomic DNA extraction according to the GeneJet Genomic DNA purification kit’s protocol (Thermo Fisher Scientific, Waltham, MA, USA). The concentration and quality of extracted DNA was determined using Nanodrop ™ Lite spectrophotometer (Thermo Scientific, Wilmington, DE, USA), Qubit dsDNA BR assay (Thermo Scientific, Wilmington, DE, USA) and 1% (v/w) agarose gel electrophoresis. The genomic DNA was then subjected to sequencing via Illumina HiSeq. 2500 and MinION.

### Illumina Sequencing

DNA was fragmented using Covaris to a targeted size of 350 bp and upon adapter ligation, a library containing fragments of 470 bp was generated. The library size was determined using Bioanalyzer high sensitivity DNA chip (Agilent, CA, USA). Library was prepared using NEBNext Ultra DNA Library Prep Kit for Illumina (NEB, MA, USA) and paired-end sequenced.

### Oxford Nanopore MinION Sequencing

Approximately 500 ng genomic DNA was used to build a DNA library using a Rapid Sequencing Kit (SQK-RAD004) (ONT, Oxford, UK) as described by the manufacturer. MinKNOW software version 2.0 (ONT, Oxford, UK) was used to perform a quality check on the flow cell before the DNA library was loaded. Sequencing was performed on MK1B (MIN-101B) MinION platform with a FLO-MIN 106 R9.4 (SpotON) flow cell according to the manufacturers’ instructions. Raw sequence reads were basecalled real time using MinKNOW, producing Fastq format data.

### Hybrid genome assembly

The FastQ format data obtained from Illumina and MinION sequencing was subjected to genome assembly using Unicycler version 0.4.3 with default parameters.

### Genome annotation

The assembly was annotated with Prokka^15^. Genome-wide COG functional annotation was performed using eggNOG mapper with DIAMOND mapping mode, which is available in version 4.5.1^16,17^. Following this, the amino acid sequences were subjected to KEGG analysis via KAAS for pathway mapping. Prophages and genomic islands were also identified using PHASTER^18^ and IslandViewer 4^19^.

## Data Records

Sequencing raw reads obtained from Illumina and Nanopore MinION runs have been deposited in the NCBI Sequence Read Archive under SRP185601 (accessible at https://identifiers.org/ncbi/insdc.sra:SRP185601)^20^. All predicted genes and their functional annotations are provided in GenBank (Accession number: NZ_CP035765)^21^. The circular genome assembly for *S*. *paucimobilis* has been deposited in NCBI Assembly under GCA_003314795.2^22^, and the whole project is at BioProject under PRJNA478628 (https://identifiers.org/bioproject:PRJNA478628).

## Technical Validation

**Fig. 2:**
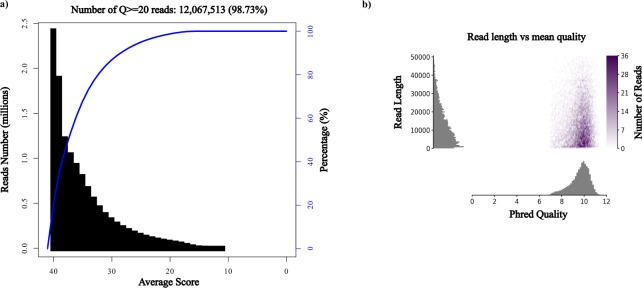
Phred analysis of Illumina and MinION reads for the *Sphingomonas paucimobilis* AIMST S2 strain genome. (**a**) Q scores for Illumina reads. (**b**) Q scores for MinION reads.

FaQCs was used to obtain the sequencing statistics and Q scores of Illumina short-reads, while Pauvr was used to obtain the same for MinION sequencing (Table 2). Illumina sequencing yielded paired-end reads of ~150 bases with more than 98% reads possessing Phred scores (Q scores) above 20 (Fig. 2a), when quality screening was performed with FaQCs. MinION reads were also of high quality, as shown in Fig. 2b.

**Table 2:** Basic statistics of Illumina and MinION sequencing.

	Illumina	MinION
Number of reads	6,111,374	11,688
Mean Length	150	14,176
Maximum Length	150	112,765
N50	—	22,110
Number of reads >10,000 bp	—	6,296 (~54%)

The hybrid genome assembly performed with the reads provided a complete, circular genome of *S*. *paucimobilis*, containing 4,005,505 bases, with an overall GC content of 65.73%. The sequencing coverage based on raw reads was 446.6×. A total of 3,612 coding sequences (CDS), 56 tRNAs, 1 tmRNA and 1 CRISPR array were identified. Three identical ribosomal operons were identified.

**Fig. 3:**
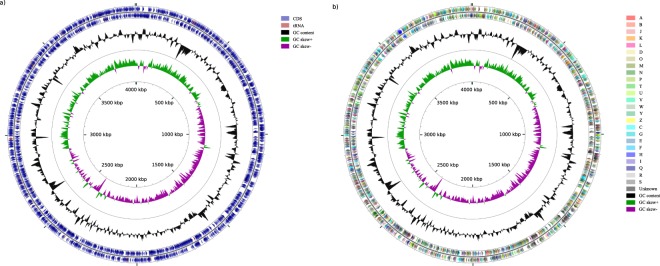
Circular map of *S*. *paucimobilis*. (**a**) Circular representation of genome with basic features including CDS and tRNA distributions. (**b**) Circular representation of genome based on COG classification.

Figure 3 illustrates the circular genome of *S*. *paucimobilis* plotted using CGView^23^.

Several levels of validation were performed to refine the hybrid assembly and check for completeness and the quality of genes predicted. Pilon refines the assembly using short reads during the final stage of assembly in Unicycler, by detecting and correcting single base differences, small and large indels or block substitution events. The present hybrid assembly was polished twice by Pilon with no changes in the assembly, suggesting an accurate assembly.

The completeness of the genomic data was further assessed according to Watson and Warr (2019)^24^. A DIAMOND blast against the UniProt TREMBL database showed that 99.1% of the genes predicted in the genome had more than 90% coverage to its top hit, suggesting good quality assembly and annotation was generated.

Among these, approximately 32 genes were shown to be involved in xenobiotic degradation (Table 3).

**Table 3:** Gene clusters involved in xenobiotic degradation.

Pathway	Number of genes involved
Benzoate degradation	8
Aminobenzoate degradation	3
Chloroalkane & chloroalkene degradation	2
Chlorocyclohexane & chlorobenzene degradation	1
Xylene degradation	1
Ethylbenzene degradation	1
Styrene degradation	1
Caprolactam degradation	4
Atrazine degradation	3
Dioxin degradation	1
Drug metabolism - other enzymes	7

Interestingly, one of the key genes responsible for organophosphate biodegradation, glutathione S-transferase, *gst* was identified in the analysis. *gst* has previously been said to detoxify xenobiotics by catalyzing the nucleophilic conjugation of reduced tripeptide glutathione (GSH; γ-Glu-Cys-Gly) into hydrophobic and electrophilic substrates^25,26^.

Apart from genes involved in chlorpyrifos and other xenobiotic biodegradation, several genes related to plant-growth promoting factors were also identified in the genome. This includes several genes in auxin biosynthesis, alkaloid biosynthesis and nitrogen metabolism. Auxin plays a significant role in promoting stem elongation^27,28^, while alkaloid plays an important role in plants by preventing insects from eating them^29^. Genes involved in nitrogen metabolism like nitrate reductase, on the other hand, is responsible in reducing nitrate to nitrite for the production of protein in most crop plants, as nitrate is the predominant source of nitrogen in fertilized soils^30–32^.

Characterization of the complete genome of *S*. *paucimobilis*, identification of potential chlorpyrifos-degrading gene, *gst* and an array of genes coding for plant-growth promoting factors opens an avenue to more studies on bioremediation and its’ potential use as an effective microorganism in bioremediation and agriculture.
